# Violence and self-reported health: does individual socioeconomic position matter?

**DOI:** 10.5249/jivr.v4i2.122

**Published:** 2012-07

**Authors:** Rocio Winnersjö, Antonio Ponce de Leon, Joaquim F Soares, Gloria Macassa

**Affiliations:** ^a^Department of Public Health Sciences, Division of Social Medicine, Karolinska Institute, SE-17176 Sweden.; ^*b*^Division of Public Health Sciences, Mid-Sweden University, 85170, Sundsvall, Sweden.; ^*c*^Departmento de Epidemiologia, Instituto de Medicina Social, Universidade do Estado de Rio de Janeiro, Brazil.; ^*d*^ Department of Occupational and Public Health Sciences, University of Gävle, 80176 Gävle, Sweden.

## Abstract

**Background:**

Violence is a major public health problem. Both clinical and population based studies shows that violence against men and women has physical and psychological health consequences. However, elsewhere and in Sweden little is known of the effect of individual socioeconomic position (SEP) on the relation between violence and health outcomes.

**Objective:**

This study aimed to assess the effect of individual SEP on the relation between violence and three health outcomes (general health, pain and anxiety) among women in Stockholm County.

**Methods:**

The study used data from the Stockholm Public Health Survey, a cross-sectional survey carried out in 2006 for the Stockholm County Council by Statistic Sweden. 34 704 respondents answered the survey, the response rate was sixth one percent. Analyses were carried out using descriptive statistics and logistic regression analysis in SPSS v.17.0.

**Results:**

Individual SEP increased the odds of reporting poor health outcomes among victimized women in Stockholm County. Regarding self-reported health women in low-SEP who reported victimization in the past twelve months had odds of 2,36 (95% CI 1.48-3.77) for the age group 18-29 years and 3.78 (95% CI 2.53-5.64) for the age group 30-44 years compared with women in high-SEP and non-victim. For pain the odds was 2,41 (95% CI 1,56-3,73) for the age group 18-29 years and 2,98 (95% CI 1,99-4,46) for women aged 30-44 years. Regarding anxiety the age group 18-29 years had odds of 2,53 (95% CI 1,58-4,03) and for the age group 30-44 years had odds of 3,87 (95% CI 2,55-5,87).

**Conclusion:**

Results showed that individual SEP (measured by occupation) matters to the relationship between violence and health outcomes such as general self-reported health, pain and anxiety. Women in lower SEP and experienced victimization in the past twelve months had increased odds of reporting poorer self-rated health, pain and anxiety compared to those in higher SEP with no experience of victimization. However, further exploration of the relationship between poverty, individual SEP is needed using other Swedish population samples.

## Introduction

Violence is a major public health problem for countries all over the world.^1^Both clinical and population based studies shows that violence against men and women has physical and psychological health consequences.^2-7^This is also true for Sweden, where several studies have found associations between victimization and physical and psychological health outcomes, particularly in women.^8-12^

Although violence is a much bigger problem in low and middle-income countries^13^it is an increasing problem in high-income countries to e.g. England and Sweden.^14,15^

Victims of violence are more vulnerable to a wide range of mental and physical health problems. Violence against women (IPV) has both acute and long-term negative health consequence e.g. poor health status, poor quality of life, injury, gynaecological symptoms or condition, depression, chronic pain, post-traumatic stress disorder, substance use.^2,4^ Depression and post-traumatic stress disorder are the most prevalent mental-health consequences of IPV.^2^ Furthermore, in industrialised countries alcohol and drug abuse are the other mental-health problem most frequently seen.^2^

An Italian study which investigated women who attended family practice, found that women who had experienced violence were more likely to be psychological distressed, evaluate their health as bad and take psychoactive drugs.^7^ In the Italian study, women who had experienced any type of violence from any perpetrator had odds of 4, 44 (2.17-9.10) for evaluated their health as poor compared with women without victimization.^7^ Another study, carried out in US, found that IPV victimization was associated with increased risk of current poor self-perceived heath, depressive symptoms, substance use, developing a chronic disease, chronic mental illness and injury.^4^

In Sweden, violence has increased in the latest 24 years; from approximately 6 percent to 8 percent among the population aged 16 years old to 74 years old and threat stands for the most of the increasing.^15^ The prevalence of victimization is high among men aged 16 to 35 years of age, single women and some occupational groups.^15^ In addition the patterns of violence differ by sex. For instance the National Board of health and Welfare reported that men were often victims from occasional and unsystematic violence outside the home and by an unknown perpetrator^12^ while violence against women is systematic, happens in the home and by a partner or ex partner.^11^ Also it has been found that violence in the workplace has increased in recent years.^16^

In Stockholm County, the frequency of violence is higher than in the rest of the nation.^11^ The age-pattern and gender pattern of violence in the County is similar to the rest of Sweden were male and female victims of violence are often of younger ages.^9,12^ As in other western countries, in Sweden studies have reported a relationship between interpersonal violence and health outcomes. For instance a study by Lundgren et al., 2001, found that women who experienced violence in general had higher mental and physical problems than women who where non-victimized, especially those who experienced threat.^11^ Furthermore in a population-based study carried out in the northern region of Sweden showed that both men and women who were victims of violence reported poor self- assessed health, anxiety, tiredness, sleep problems and pain.^8^

Studies, which have investigated the relation between violence and socioeconomic position, have so far provided mixed results. For instance in Canada, a population based study among men and women regarding IPV, reported that victimization was associated with low levels of income.^17^ In USA and Norway other studies have found associations between being victim of IPV and low levels of education, occupation and income.^18,19^ However, others have found no association between education and income and IPV in women.^20^ However, few studies have investigated the impact of socioeconomic position in the relationship between violence and health outcomes.

It can be hypothesised that individual socioeconomic position: (a) increases the likelihood of poor health outomes among victimized women and (b) it interacts with victimization to produce a high risk of poor health. 

The objective of this study was to investigate the effect of individual socioeconomic position on the relationship between violence and three health outcomes (general health, pain and anxiety) among women in Stockholm County.

## Methods

**Data and sample description **

This study uses data from the 2006 Stockholm County Public Health Survey (SCPHS). It is a cross sectional survey based on a self-administered postal questionnaire. It is a randomly stratified sample (by area) and the initial sample was 57 009 persons 18-84 years of age living in Stockholm County. A total of 34 707 individuals returned the questionnaire which gave a response rate of 61 percent. Questions were asked about health, long-stand illness, self reported specific illness according to questions about certain diseases or symptoms of diseases, housing, leisure and social relations, political activity, safety, security, violence, economy, employment and work environment. The survey was conducted by Statistic Sweden for the Stockholm County Council. More detail about the survey can be found elsewhere.^21,22^

**Specification and measurement of variables**

**Violence**

Measures of violence was assessed using the following two questions, “Have you in the past 12 months been exposed to physical violence?” and the second, “Have you in the past 12 months been exposed to threat of violence that was so severe that you got afraid?” In this study, women who answered yes to either one or both questions regarding violence were regarded as victims of violence. 

**Health status**

Three indicators of health were used. Participants were asked: “How do you evaluate you general health status? ” Possible answers were: excellent; good, fairly; bad or very bad. To be in line with prior research,^3,7^ a dichotomous variable was created, dividing those with good health (very god or god) from the others. 

Second, current self-rated pain was assessed by using the question: “Which statement does best describe your health status today: pain/discomfort”. Possible answers were: I do not have any pain or discomfort; I have moderate pain or discomfort and I have serious pain or discomfort. A dichotomous variable was created dividing those with pain (moderate pain and serious pain) from those without.

Thirdly, anxiety/physiological distress was assessed by using the question: “Which statement does best describe your health status today, anxiety/physiological distress”. Possible answers were: I have no anxiety or physiological distress; I have anxiety or physiological distress of some measure and I have extremely anxiety or physiological distress”. Again a dichotomous variable was created to distinguish those with anxiety or physiological distress (anxiety in some measure and extremely anxiety) from those without.

**Socioeconomic position**

Socioeconomic position (SEP) is a commonly used concept in health research and it refers to the social and economic factors that influence what position individuals or groups hold within the structure of a society.^23,24^ There is no single best indicator of SEP but the traditional individual measures of SEP are income, education and occupation.^23,24^ Although these indicators have limitations, a large amount of epidemiological evidence has shown the importance of these factors as health determinants.^24^ In this study, we use occupation as a measure of socioeconomic position. In Sweden occupation is often used as an indicator of SEP and it is primarily based on the occupation and the normally requiring education level that the occupation requires.^25^ In addition, classification of occupation categories is ruled by the Nordic Classification of Occupations, which is based on the International Standard Classification of Occupation.^25^ Respondents in the SCPHS were asked about their own current occupation or their main occupation when working and the type of job assignment. Respondents were divided into six socioeconomic groups based on the occupational information they gave: higher non-manual; intermediate non-manual; lower non-manual; skilled manual, unskilled manual and own entrepreneurs. For the analysis the group own entrepreneurs will be omitted because it is a socioeconomically heterogeneous group and also to be in line with earlier research.^26^

**Education level **

Education level was assessed from the Statistics Sweden’s LISA database from 2004 and was grouped by the Swedish educational nomenclature SUN 2000 (old version).^27^ The original variable is classified into seven groups from lowest to highest: primary school shorter than nine years; primary school nine or ten years; upper secondary school maximum two years; upper secondary school more then two years and maximum three years; higher or further education shorter than three years; higher or further education three years or more; post-graduate study. For the analysis three groups were created: primary school or similar; secondary school/similar and university/similar. 

**Social support**

Social support was measured by using the question “Do you have one person or more that can give you support when you have personal problems or crisis in your life?” Possible answers were: yes, always; yes, most of the time; no, not most of the time and no, never. A dichotomous variable was created dividing those with social support (yes always and most of the time) from those without. 

**Work strain**

Work strain was measured by two separate variables, demand and control at work. The demand variable was assessed using the question “Do you have enough time to do your work assignments?” and the control variable was assessed using the question “Do you have the freedom to decide how the work is being done?” Possible answers on both of the questions were: yes, always; yes, most of the time; no, not most of the time and no, never. For each question a dichotomous variable was created dividing those with demand at work or control at work (yes always and most of the time) from those without. 

**Age**

Violence vary by age, young people are at more risk for violence than old.^1,9,12^ For this reason and to be in line with previous studies in Sweden regarding violence and health two age groups were created 18-29 years and 30-44 years.^8^

**Statistical analysis **

The data analysis was performed using SPSS V. 17,^28^ by using descriptive statistics and logistic regression analysis. Logistic regression analyses were carried out in three steps, first to estimate the odds ratios of victimization SEP; second, the odds ratio of the three health outcomes by SEP. 

The third step consisted in logistic regression analysis to assess the relationship between a composite variable, which was constructed using occupation and violence and the three health outcomes (general self-reported health, pain and anxiety). To this end, occupational grade was dichotomized on the basis of occupational hierarchy into high-SEP (non-manual workers) and low-SEP (manual workers). Violence was dichotomized as Victim and non- Victim. The dichotomised exposure variables, SEP and violence were combined in the following way: (1) low-SEP and Victim (2) high-SEP and Victim (3) low-SEP and non-Victim (4) high-SEP and non-Victim. Results are presented as OR with 95% confidence intervals. 

The fourth step in order to test if there was interaction of the two exposures (socioeconomic position and violence) the Rothman’s model for analysis of biological interaction or synergism was applied. The model assesses if there are cases occurring only in the presence of joint exposures (in this study, victimization and low socioeconomic position), in other words whether there is a departure from additivity of effects.^29^ The synergy (S) index with 95% CI was used.^30^

**Figure:**
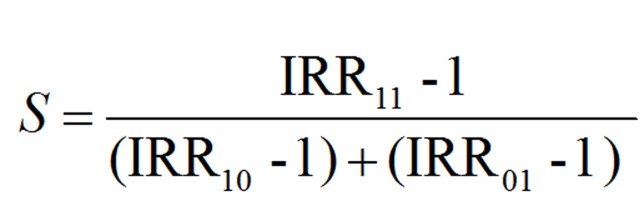


IRR indicates the incidence rate ratios, with IRR00 is the unexposed denominator, IRR_10_ and IRR_01_ are for those with one exposure, either being victim or having a low SEP, IRR_11_ is for those with both exposure. If the value exceeds 1.0 the index indicates synergy and if the value is below 1.0 it indicate antagonism. 

## Results

**Socioeconomic position, violence and health outcomes**

Self-reported victimization was 9.4% among women in the age group 18-29 years and 6.9% among those in the age group 30-44 years. The overall descriptions of the variables included in the analysis are presented in Table 1

**Table:** Table 1: ** Descriptive data of the women 18-44 years old from the SCPHS 2006**

Variable	N=3217	%	N=5677	%
Age	18-29		30-44	
Violence				
Yes	303	9.4	393	6.9
No	2877	89.4	5212	91.8
Missing	37	1.2	72	1.3
Socioeconomic position				
Unskilled manual worker	816	25.4	634	11.2
Skilled manual worker	320	9.9	541	9.5
Lower non manual worker	464	14.4	884	15.6
Intermediate non manual worker	442	13.7	1587	28.0
Higher non manual worker	298	9.3	1264	22.3
Own entrepreneur	108	3.3	336	5.9
Missing	770	23.9	431	7.6
General health status				
Excellent	772	24.0	1486	26.2
Good	1749	54.4	2957	52.1
Fairly	564	17.5	980	17.3
Bad	87	2.7	175	3.1
Very bad	20	0.6	32	0.6
Missing	25	0.8	48	0.8
Pain and/or discomfort				
No pain/or discomfort	2149	66.8	3329	58.6
Moderate pain and/or discomfort	885	27.5	1958	34.5
Serious pain and/or discomfort	49	1.5	153	2.7
Missing	135	4.2	238	4.2
Anxiety				
No anxiety or physiological distress	1618	50.3	3417	60.2
Moderated anxiety or physiological distress	1338	41.6	1917	33.8
Severe anxiety or physiological distress	122	3.8	121	2.1
Missing	138	4.3	223	3.9

In the bivariate analysis occupational level was related with being victim of violence among women in both age groups. The highest odds were found for skilled manual workers. Compared with higher-non-manual workers, skilled manual workers aged 18-29 years had the odds of 2,6 (CI 1,42 to 4,77) to be victims of violence and for the group 30-44 years old the odds was 4,4 (CI 2,95 to 6,57) (see Table 2 respectively 3). In addition unskilled manual workers aged 18-29 years had odds of 2,37 (CI 1,37 to 4,12) and those aged 30-44 years had odds of 3,69 (CI 2,48 to 5,49) for being a victim of violence compared with higher-non-manual women (see Table 2 respectively 3).

**Table:** Table 2: **Odds ratios of self reported health and violence by occupation, age group 18-29 years, SCPHS, 2006.**

Variable	General Health		Pain		Anxiety		Violence	
	OR	95% CI	OR	95% CI	OR	95% CI	OR	95% CI
Socioeconomic position								
Unskilled manual worker	3,97	2,54 to 6,20	1.54	1,13 to 2,09	1,44	1,09 to 1,89	2,37	1,37 to 4,12
Skilled manual worker	3,85	2,36 to 6,27	1,61	1,13 to 2,31	1,36	0,98 to 1,88	2,6	1,42 to 4,77
Lower non manual worker	3,12	1,94 to 5,00	1,31	0,94 to 1,83	1,48	1,10 to 1,99	1,86	1,02 to 3,38
Intermediate non manual worker	2,06	1,26 to 3,36	1,14	0,81 to 1,61	0,80	0,59 to 1,09	1,94	1,06 to 3,52
Higher non manual worker	1		1		1		1	

* All analyses are adjusted for education level

**Table:** Table 3: **Odds ratios of self reported health and violence by occupation age group 30-44 years, SCPHS, 2006.**

Variable	General health		Pain		Anxiety		Violence	
	OR	95% CI	OR	95% CI	OR	95% CI	OR	95% CI
Socioeconomic position								
Unskilled manual worker	4,12	3,26 to 5,22	2,59	2,12 to 3,17	2,24	1,83 to 2,74	3,69	2,48 to 5,49
Skilled manual worker	3,36	2,61 to 4,31	2,53	2,05 to 3,14	1,68	1,36 to 2,09	4,40	2,95 to 6,57
Lower non manual worker	1,97	1,56 to 2,49	1,62	1,34 to 1,95	1,54	1,28 to 1,86	1,93	1,28 to 2,92
Intermediate non manual worker	1,39	1,12 to 1,72	1,36	1,15 to 1,59	1,34	1,14 to 1,58	2,15	1,49 to 3,10
Higher non manual worker	1		1		1		1	

* All analyses are adjusted for education level

Occupation was also associated with general self-rated health for women aged 18-29 years and 30-44 years. For the age group 18-29 years unskilled manual workers had the odds of 3,97 (CI 2,54 to 6,20) compared with professionals of rating their health as bad and skilled non-manual workers odds of 3,85 (CI 2,36 to 6,27) (seeTable 2). In addition, in the age group 30-44 years unskilled manual workers had odds of 4,12 (CI 3,26 to 5,22) compared with higher non-manual workers (see Table 3).

For women both age groups there were an association between occupation level and reporting pain or discomfort. Unskilled manual workers aged 18-29 had the odds of 1,54 (CI 1,13 to 2,09) compared with higher non-manual workers and skilled manual workers had the odds of 1, 61(1,13 to 2,31) compared with higher non-manual workers (see Table 2). In addition women aged 30-44 years with unskilled manual work had the odds of 2,59 (CI 2,12 to 3,17) and for skilled manual workers the odds was 2,53 (CI 2,05 to 3,14) (see Table 3).

In relation to anxiety or physiological distress and among women aged 30-44 years there was a statistical association with occupational grade. Unskilled manual workers had the odds of 2,24 (CI 1,83 to 2,74) compared with higher non-manual workers (see Table 3). Furthermore skilled non-manual workers had the odds of 1,68 (CI 1,36 to 2,09) compared with higher non-manual workers (see Table 3). For the women aged 18-29 compared with higher-non-manual workers unskilled manual workers had the odds of 1,44 (CI 1,09 to 1,89) for reporting having anxiety (see Table 2).

**The role of socioeconomic position in the relationship between violence and health outcomes**

In the regression analysis there was a relationship between low-individual socioeconomic position (SEP) and victimization and self- reported health status among women aged 18-29 and 30-44 years of age. Compared to women of high SEP and non-victims, women aged 18-29 years of age with low-SEP and victims of violence experienced odds of poor self-reported health of 2.36 (CI 1.48 to 3.77 (see Table 4). In addition, women aged 30-44 years with low socioeconomic status and victimized had odds of 3.78 (CI 2,53 to 5.64) (see Table 5).

**Table:** Table 4: **Association of violence and socioeconomic position (occupation grade) with three health outcomes, women aged 18-29 years, SCPHS, 2006.**

Variable	General Health		Pain		Anxiety	
	OR	95% CI	OR	95% CI	OR	95% CI
SEP and Violence						
Low SEP and victim	2,36	1,48 to 3,77	2,41	1,56 to 3,73	2,53	1,58 to 4,03
High SEP and victim	1,84	1,09 to 3,12	1,60	0,99 to 2,56	2,76	1,67 to 4,55
Low SEP and non-victim	1,27	0,96 to 1,68	1,22	0,96 to 1,55	1,16	0,93 to 1,45
High SEP and non-victim	1		1		1	
Hosmer-Lemeshow goodness-of-fit chi-square statistics	0,10		0,68			0,58

* All analyses are adjusted for education level, social support, work demand and work control.

**Table:** Table 5: **Association of violence and socioeconomic position (occupation grade) with three health outcomes, women aged 30 to 44 years old, SCPHS, 2006.**

Variable	General Health		Pain		Anxiety	
	OR	95% CI	OR	95% CI	OR	95% CI
SEP and Violence						
Low SEP and victim	3,78	2,53 to 5,64	2,98	1,99 to 4,46	3,87	2,55 to 5,87
High SEP and victim	2,20	1,53 to 3,16	1,58	1,15 to 2,17	2,58	1,86 to 3,58
Low SEP and non-victim	1,93	1,56 to 2,38	1,50	1,25 to 1,79	1,28	1,06 to 1,55
High SEP and non-victim	1		1		1	
Hosmer-Lemeshow goodness-of-fit chi-square statistics	0,25		0,31		0,37	

* All analyses are adjusted for education level, social support, work demand and work control.

In relation to pain, women 18-29 years with low SEP and victim of violence had odds of 2,41 (CI 1,56 to 3,73) for reporting pain or discomfort compared with women with high SEP and non-victim (see Table 4). In addition women 30-44 years of age who had low SEP and suffered violence had odds of 2,98 (1,99 to 4,46) compared to those with high SEP and without victimized (see Table 5).

Furthermore, with regard to Anxiety, women in the younger group who had low SEP and victimized had odds of 2,53 (1,58 to 4,03) compared with those with high SEP and non-victim (see Table 4). Women in the older age group hade odds of 3,87 (2,55 to 5,87) for reporting anxiety or downheartedness compared with women compared with those with high SEP and without victimized (see Table 5).

In order to access the appropriateness and adequacy of the models fit, the Hosmer-Lemeshow goodness of fit test was performed. Results showed that the fit of the two models was satisfactory, with p greater than 0.05 (see Table 4 and 5).

Interaction analysis showed that there was no interaction between individual socioeconomic position and victimization in the risk of poor health outcomes. The Rothman’s synergy index was less than one (results not shown).

## Discussion

Results of this study have shown that manual workers had higher odds of victimization than non-manual workers for both age groups. This finding is in line with other studies. In a Swedish national study found that women exposed to physical violence with manual occupation had the odds of 1,63 (CI 1,27-2,09) compared with women in higher non-manual occupation.^31^ Although we have not found any more studies with the same socioeconomic indicator (occupational level) in relation violence victimization the current study supports other studies that found that low SES/SEP have associations with violence victimization. For example a Norwegian study found that low economic status was one of the factors most frequently associated with IPV^19^ and a Swedish study found that women that had problems getting 14 000 SEK in a week hade odds of 2,6 (CI 2,4-2,7) of being victims of violence than women without this financial problem.^8^ The findings regarding SEP and victimization in the current study may help explain by the social structure theory. The structural approach to violence is that it is unevenly distributed in the social structure.^32^ Violence is more common among those in lower socioeconomic position and is seen as a result of different distribution of the main causes of violence (stress and frustration).^32^

Results of this study also showed a similar pattern of social gradient (by occupation) as regarding the three health outcomes (self rated health, pain and anxiety) for both age groups. Other studies have found that individuals in low occupational positions reported poorer self-rated health, anxiety and pain.^33,34^

In addition this study found that women being victims of violence (regardless of SEP) reported their health as poorer then non-victimized women, this finding are in line with other studies.^4,7,8,19^

**Why addressing the role of individual socioeconomic position on the relationship between violence and health outcomes?**

The majority of available studies in Sweden and elsewhere in the developed world addresses separately the relationship of SEP and violence, SEP and health outcomes and victimization and health. However, as mentioned above, this study moved beyond to hypothesize that socioeconomic position may increase the risk of women experiencing poor health outcomes or that socioeconomic position interacted with victimization to produce poor health.

These two hypotheses were put forward taking into consideration that violence is a major life stressor that affects the individual negatively on one hand and low socioeconomic position is another well-known stressor, which in addition can bring an even poorer health outcome. The assumption was that there might be a compound effect or a synergic interaction between low SEP and victimization. In case of interaction that would mean that there was a joint effect of the two exposures, which would exceed the independent effects of socioeconomic position (by occupation) and victimization alone. However, as mentioned above there was no positive interaction between the two exposures, at least in the sample under study.

In accordance with the hypothesis that individual socioeconomic position increased the odds of poor health outcomes this study found that compared with women with high SEP and non-victims women aged 18-29 and 30-44 years of age in low SEP and victimized had higher odds of reporting poor general self-rated health, pain and anxiety (see Table 4 and 5). In addition for those in low SEP and being victimized the age groups 30-44 years of age had higher odds then women aged 18-29 years (see Table 4 and 5).

Social groups differ when it comes to levels of stress, deprivation, frustration and the resources to deal with these stresses.^32^ It is argued that people in higher SEP have access to more resources and can cope better with the stressors.^32,35^ Those from lower SEP are more strongly affected emotionally by negative life events than those in higher SEP.^35^ The differences in access to resources may explain why those in the low SEP and being victim of violence have higher odds for reporting their health as poor than those with high SEP and non-victim of violence.

Others argue that violence increases the risk for poverty and unemployment^36,37^ which in turn have negative effects on health.^38^ For instance, in a study of welfare recipients Tolman and Rosen reported that recent victims of relationship violence had significantly higher rates of depression, anxiety, posttraumatic stress disorder, poor self-rated health, and a drug and alcohol dependence than women with no violence in their lives.^39^People living in poverty experience daily stressors in meeting the basic needs such as obtaining food, shelter, transportation or clothing and keeping themselves and their families safe.^40^ In addition poverty can make the daily lives of women and children’s more dangerous and make them more dependent on others for survival and therefore less able to recognise their own victimization or to seek help when victimized.^40^ It is suggested that in high-income countries, poverty (relative poverty) and violence play a kind of toxic dance in women’s lives. Poverty marginalizes women, increasing their risk of victimization, while violence also isolates women, as the mental and physical effects grind away at women’s sense of well-being, limiting what is possible.^41^

In a review of the role of social inequalities in violence against women Humphrey stated that the evidence of the impact of violence on health was uncontested.^38^ However she argued that the extent to which sources of inequality such as poverty, disability and ethnicity create vulnerabilities to violence for women were more complex and needed to be addressed.^38^ This argument can be held for not just violence against women but for interpersonal violence among men. 

In Sweden, violence is a public health problem that is increasing.^15^ It has been showed that there is a socioeconomic difference in the burden of disease in Sweden.^26^ but not much is known about violence and inequality. Some suggest that the relation between inequality and health is very similar to the association between inequality and violence.^42^Therefore, there is a need to disentangle the role that poverty and social class bears in the experiences of victimization among Swedish women and men alike. 

**Strengths and Limitations **

One of the strengths of this study is that the collection of SCPHS data was carried out by Statistics Sweden on behalf of Stockholm County Council. In addition, the study uses self-rated health as one of the health outcomes. Self-rated health has been reported as a valid predictor of morbidity and future health care use and are therefore relevant for analyzing health related victimization. ^43,44^

However, this study is not without limitations. The data used is cross-sectional therefore causality may be difficult to establish; violence is a sensitive subject and it is suggested that there is an under reporting of violence in surveys;^45^ the SCPHS had only two questions about violence physical violence and threat of violence leaving other types of violence e.g. verbal, sexual and emotional out; the variable “violence” was a merged variable due to small numbers. However as Rutheford et al argues, each type of violence has a specific set of factor and determinants but never the less these are common across different types of violence.^46^ In addition no level of severity can be drawn from the data. Furthermore, the data did not allow the use of the Karasek and Theorell scale of work demand and control. That scale would have captured more dimensions regarding work strain. Finally, it is possible that other variables, which were not included in the study, may help explain the relationship between violence, SEP and the three health outcomes.

## Conclusion

Results of this study showed that individual SEP (measured by occupation) matters to the relationship between violence and health outcomes such as general self-reported health, pain and anxiety. Women who were in lower SEP and experienced victimization in the past twelve months had the greatest odds of reporting poorer self-rated health, pain and anxiety compared to those in higher SEP with no experience of victimization. However, further exploration of the relationship between poverty, individual socioeconomic position and victimization is needed using other Swedish population samples.
